# The Evaluation of the Cervical Marginal Sealing of Direct vs. Indirect Composite Resin Restorations in MOD Cavities

**DOI:** 10.3390/dj12040092

**Published:** 2024-04-03

**Authors:** Bianca Tiron, Norina Consuela Forna, Ionuț Tărăboanță, Simona Stoleriu, Claudiu Topoliceanu, Mihaela Sălceanu, Răzvan Brânzan, Gianina Iovan

**Affiliations:** Faculty of Dental Medicine, Grigore T. Popa University of Medicine and Pharmacy, 16 Universitatii Str., 700115 Iasi, Romania

**Keywords:** inlay, nanohybrid composite resin, etch-and-rinse, self-etch, marginal sealing

## Abstract

Introduction: The aim of this in vitro study was to compare the quality of marginal sealing at the cervical margins of indirect and direct composite resin restorations in mesio-occluso-distal (MOD) cavities. Material and method: MOD preparations were performed on 30 extracted teeth. The mesial cervical margin of each tooth was relocated using a flow composite resin (Enamel Plus HRi Flow, Micerium, Avegno, GE, Italy), then the samples were divided into three groups. In group A, the cavities were directly restored using a nanohybrid composite resin (Miris 2 Coltène Whaledent, Altstaetten, Switzerland) and a universal adhesive (ScotchBond Universal, 3M ESPE, St. Paul, MN, USA) by the etch-and-rinse strategy, for group B, the restoration procedure was similar but the self-etch strategy was used, and the samples in group C were filled using the inlay technique. Each sample was stored for 48 h in a 2% methylene blue solution, then it was cut in a mesio-distal direction using a Struers Secotom 50 device (Cleveland, OH, USA). The marginal sealing and adhesive interface were assessed for each sample at the cervical margin by optical microscopy (OM) and scanning electron microscopy (SEM). One-way ANOVA and Bonferroni post-hoc tests were used with a significance level of 0.05. Results: Significant differences were recorded within groups A and C, between mesial and distal margins (*p* = 0.02 in group A and *p* = 0.043 in group C). Conclusions: The marginal sealing is more effective in MOD inlay restoration compared to direct restorations. Relocation of the cervical margin with flow composite resin and the use of different adhesive strategies do not improve the marginal sealing.

## 1. Introduction

As a result of the increasing aesthetic demands of patients, resin-based restorative materials have become the main option in direct and indirect restorative treatments [1]. Several studies showed that a percentage of approximately 60% of the procedures performed in dental offices is represented by cavities restoration or the replacement of old restorations [2,3]. The clinical success of an operative/restorative treatment depends on the choice of materials, their handling, and the correctness of the application technique [3]. Composite resins are mainly used due to their superior properties compared to other restorative materials, their adhesion to hard dental tissues, and as a result of their multiple application possibilities such as direct or indirect restoration of cavities, or as a cementing material [4].

Regarding the disadvantages of composite resins, they include polymerization shrinkage rated at 0.3–1.5% linear shrinkage, respectively, 1.5–3.5% volumetric shrinkage for Bis-GMA monomer-based resins, an increased wear estimated at 12–50 µm/year, and a volumetric expansion approximately 6 times higher than hard dental tissues [4].

The marginal adaptation of the restorative material to cavity walls is the main factor that can influence the longevity of the restorations and is directly related to the location, size, shape or configuration factor (C factor) of the cavity [5,6]. In complex mesio-occluso-distal (MOD) cavities, the extension of the restoration and the high load exerted on it can lead to failure [7]. At the same time, the structure, properties, and composition of composite resins as well as the type of adhesive strategy used influence the bonding strength to hard dental tissues [4,8].

The major clinical consequences of adhesive bond damage consist of the occurrence of postoperative hypersensitivity, fractures in the restorative material or cavity walls, or the occurrence of carious lesions adjacent to restorations [9].

Over time, several techniques have been tested to combat the shrinkage effect of composite resins, such as applying the material in oblique layers or delayed photoactivation also known as the “soft-start” technique, but none of these methods significantly reduced the shrinkage effect of the materials [10,11]. Similarly, the incorporation of high molecular weight monomers such as ormocers or siloranes did not lead to the appearance of resins with significantly lower shrinkage effect [12].

To eliminate the shortcomings shown by the direct dental restorations, the indirect techniques such as inlays started to be used [4,13]. Inlays are polymerized outside the oral cavity, the material being exposed to light, pressure and heat, thus achieving a much higher conversion degree of monomers compared to direct restorations that are light activated in the oral cavity and whose monomers convert in a percentage between 28 and 73% [4,14]. Inlays showed a good Young’s modulus of elasticity, as well as an efficient resistance to fracture, wear, and flexure superior to direct restorations [1,4]. However, the studies realized over time have not demonstrated which of the two restoration techniques is more advantageous [3].

The aim of this in vitro study was to evaluate and compare the marginal sealing quality at the cervical margin of composite resin direct restorations vs. composite resin inlays applied in MOD cavities. The null hypothesis of the study was that cervical marginal sealing in MOD cavities does not differ for both direct and indirect restorations.

## 2. Materials and Methods

This study was conducted in accordance with the Declaration of Helsinki and the rules imposed by the Ethics Committee of the “Grigore T. Popa” University of Medicine and Pharmacy of Iași, Romania (no. 66/07.04.2021).

### 2.1. Sample Preparation

The recommended sample size was calculated using G* Power software (Heinrich-Heine Universität Düsseldorf, Düsseldorf, Germany) version 3.1.9.7, with an effect size of 0.6 calculated on the basis of mean values and standard deviations, which is considered to be a large effect according to Cohen’s classification, an alpha value of 0.05, and a statistical power of 80%. The obtained results indicated the use of a minimum amount of 30 samples.

**Figure 1:**
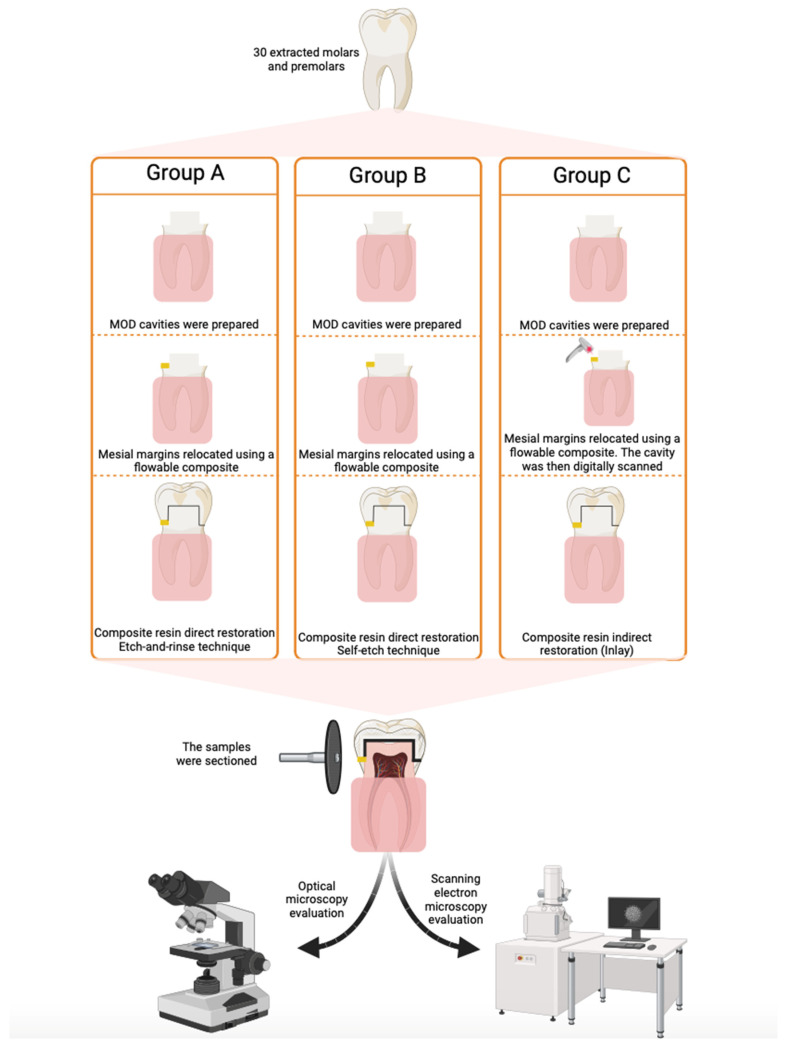
Schematic representation of the study stages.

A total number of 30 teeth (molars and premolars) extracted from orthodontic or periodontal reasons, without caries, cracks or restorations, were used and mesio-occluso-distal (MOD) cavities were prepared. The cervical margins were placed in dentin, apical to the cementoenamel junction (Figure 1). After the cavities were prepared, the teeth were stored in saline solution for 2 days. Then, the roots were embedded in cylindrical blocks made of self-curing acrylic resin to facilitate the cutting procedure. The margins of the acrylic block were placed at a minimum distance of 3 mm apical from the enamel–cement junction. The long axis of the tooth was oriented perpendicular to the surface of the acrylic block using a parallelometer (Degussa F1, DeguDent, Hanau, Germany). The mesial cervical margin of each cavity was relocated (Figure 1) using a flow composite resin Enamel Plus HRi (Micerium, Avegno, GE, Italy). Subsequently, 20 of the prepared cavities were directly restored using a nanohybrid composite resin Miris (Coltène Whaledent, Altstaetten, Switzerland) and a universal adhesive resin ScotchBond Universal (3M ESPE, St. Paul, MN, USA) using the etch-and-rinse strategy for half of the samples (n = 10) and the self-etch technique for the other half (n = 10). The application protocols for etchant and adhesive are detailed in Table 1.

The nanohybrid composite resin was applied in 2 mm layers, and each layer was light-cured for 40 s according to the manufacturers’ recommendations using an LED light-curing lamp (X-Cure LED, WoodPecker, Guilin, China) (Figure 1) with a wavelength between 385 nm and 515 nm with an LED blue light of 10 W. The other 10 teeth were digitally scanned using a Medit I700 scanner (Medit Corp. Seoul, Republic of Korea) and an Exocad Dental CAD (Exocad, Darmstadt, Germany) software, version 3.2, and then were indirectly restored. Inlay pieces were designed by an operator using Exocad Dental CAD. The obtained inlay restorations were made of Miris 2 nanohybrid composite resin and cemented using Rely X Unicem (3M ESPE, St. Paul, MN, USA) self-adhesive composite resin cement. Thus, the study samples were divided into 3 study groups (groups A, B, and C) corresponding to the restoration technique or adhesive strategy used. Cavity preparation and restoration were performed by a single operator. Each study group was then divided into 2 subgroups: distal cervical margins–Subgroup “Cerv”; mesial cervical margins–Subgroup “Flow”. The relocation of the mesial margins with flow composite was performed after an etch-and-rinse adhesive strategy was applied on the cervical dentin wall, followed by the application of the flow composite in a layer of 1 to 1.5 mm thick. A circumferential stainless steel matrix and a wooden wedge were used. The direct and indirect restorations were applied immediately after the cervical margin relocation. Details related to the composition, manufacturer, and type of the tested materials are presented in Table 2.

### 2.2. Sample Preparation for Marginal Sealing Evaluation

The samples were then submersed in a 2% methylene blue solution for 48 h, after which they were washed under a continuous stream of water for 2 min and stored for 24 h in distilled water. To evaluate the marginal sealing at dentin–material interface for both mesial and distal cavities, the teeth were sectioned in a mesio-distal direction using a Struers Secotom 50 device (Struers LLC, Cleveland, OH, USA) equipped with a 0.5 mm thick water-cooled diamond disc. At the mesial margin, the dentin-flowable composite resin interface was assessed, while at the distal margin, the interface between dentin and conventional composite was evaluated. Then, the surfaces were finished and polished using Sof-Lex finishing and polishing discs (3M ESPE, St. Paul, MN, USA).

### 2.3. Evaluation of Marginal Sealing by Optical Microscopy

The evaluation of marginal adaptation was performed by optical microscopy analysis using a Zeiss Imager microscope (a1M Carl-Zeiss, Jena, Germany) equipped with an Axiocam digital camera. The images were obtained using the AxionVisionRelease 4.7.1 software. The used magnification was of 50×, in BF field (bright field). The measurement of marginal microleakage gaps was performed using the 500/1000 µm scale provided by the AxionVisionRelease software (Figure 1).

### 2.4. Evaluation of the Adhesive Interface by Scanning Electron Microscopy (SEM)

A Scanning Electron Microscope Vega Tescan LMH II (Tescan, Kohoutovice, Czech Republic) was used to assess the morphology of the interface between the composite resin and the dental tissues. The used operating conditions were of 30 kV and 15.5 WD. The cervical margins and internal walls were analyzed in terms of integrity and microgaps formation. A gold layer of 10 nm was used to coat the samples using a LUXOR^TM^ benchtop sputter coater (ULVAC Technologies, Inc., Kanagawa, Japan).

### 2.5. Statistical Analysis

The statistical analysis of the obtained data was performed using SPSS software (SPSS Inc., Chicago, IL, USA) version 29.0. Shapiro-Wilk test was used to assess the normality of distribution of the data and the analysis was performed using One-Way Analysis of Variance ANOVA and post-hoc Bonferroni statistical tests, with a significance level of 0.05.

## 3. Results

**Figure 2:**
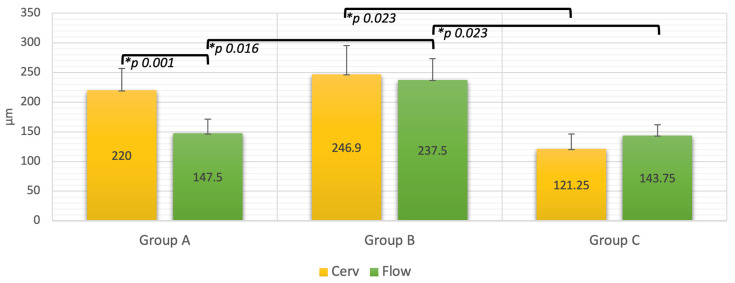
Mean values of microleakage, standard deviation (µm), and significant differences between groups. Cerv-Cervical margins; Flow-Cervical margins relocated with flow composite resin. * Statistically significant differences.

Analyzing the obtained microleakage values (µm) in each study group at the level of each evaluated margin, it can be observed that at each of the margins in group B recorded the highest values: 246.9 ± 48.3 µm at the cervical margin and 237.5 ± 36 µm at the margins relocated with flow resin. The lowest values were recorded by group C at each margin: 121.25 ± 36 µm at the cervical margin and 143.75 ± 18 µm at the margin relocated with flow resin (Figure 2).

At the cervical margin, significant differences (*p* < 0.05) were recorded between groups B and C (*p* = 0.00), while at the margins relocated with flow composite, significant differences were found between groups A and B (*p* = 0.016) and B and C (*p* = 0.023).

Within group A, significant differences were recorded between the obtained mean values of Cerv and Flow margins with a *p* of 0.001. In groups B and C, no significant differences were recorded between the mean values (*p* > 0.05).

**Figure 3:**
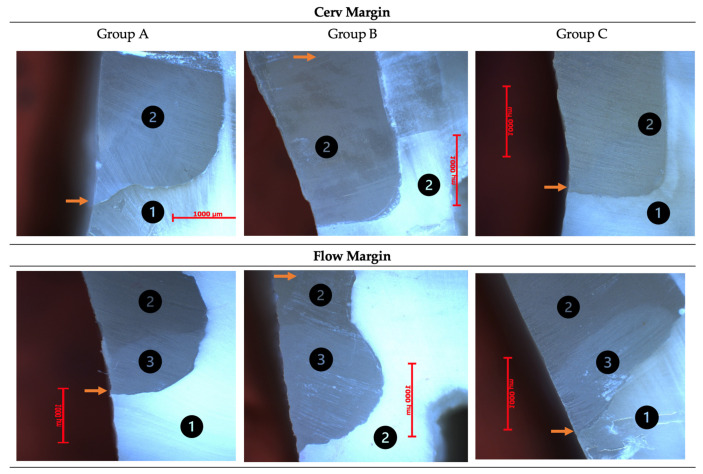
Optical microscopy images of Cerv and Flow margins of a sample in each study group. 1—dentin; 2—conventional composite resin; 3—flowable composite resin; the interface between the conventional/flowable composite resin and dentin is indicated by arrow 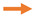.

In the figure below (Figure 3), the presented images are obtained by optical microscopy of both mesial and distal margins (Cerv and Flow) of a sample in each study group.

**Figure 4:**
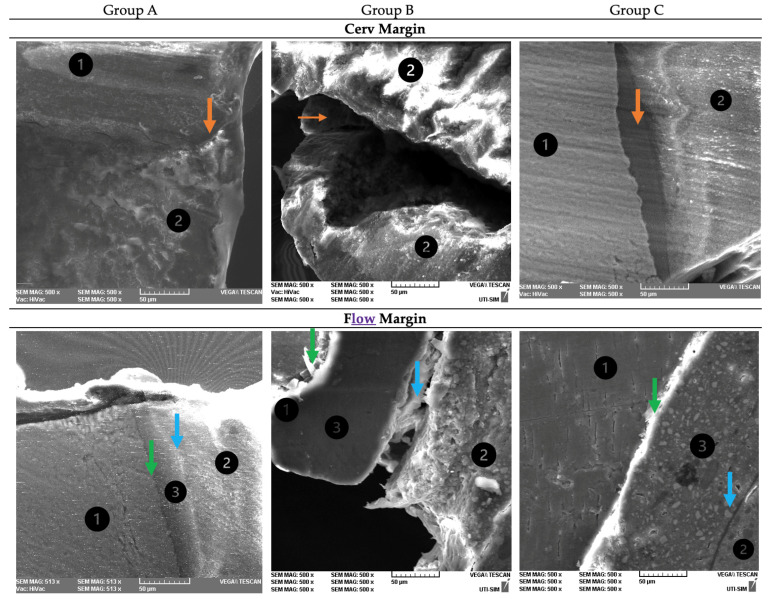
Representative scanning electron microscopy (SEM) images of cervical margins with and without flow composite margin relocation of a sample in each study group, at 500× magnification. 1—dentin; 2—composite resin; 3—flow composite; Adhesive interfaces: conventional composite-dentin: orange arrow 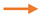; flowable composite-dentin: green arrow 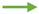; conventional composite-flowable composite blue arrow 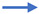.

The microleakage areas were evaluated at both cervical margins (with and without flow composite relocation). The image below (Figure 4) presents the representative scanning electron microscopy (SEM) images of both cervical margins of a sample in each group, at 500× magnification. When analyzing the cervical margins (subgroup Cerv) in group A, an intimate contact was observed between tooth walls and restorative material mediated by a thin adhesive layer. In group B, a damaged dentin–composite resin interface was observed, while in group C, an adequate marginal sealing was observed. For the cervical margins relocated with flow composite, in group A, an intimate contact was observed between tooth walls and composite resin. In group B, a large marginal gap and adhesive failure were observed between the flow composite and both dental walls and restoration material. In group C, a close contact between flow composite and both dental walls and the cementing material was noticed.

## 4. Discussion

The present in vitro study is relevant from a clinical point of view, given that the decision to treat by direct or indirect restoration can influence time, financial resources or the patient’s life quality.

The marginal adaptation of restorative materials to cavity walls is one of the most important factors that influence the longevity of composite resin restorations [15]. The disadvantages of composite resins are mainly represented by polymerization shrinkage and stress that may lead to an inadequate sealing of the cavity, thus increasing the possibility to develop secondary carious lesions, dentinal hypersensitivity or marginal staining [16]. For this reason, numerous attempts have been made to combat these shortcomings by introducing new types of monomers with different chemical structures, changing the proportions of the filler particles, preheating of the materials or applying the restorations through indirect or semi-direct techniques such as inlays, onlays or overlays [4,17]. However, none of these techniques showed significant reduction in the marginal microleakage values [18]. Microleakage can be associated with an insufficient bonding of the adhesive resin to hard dental tissues such as enamel (especially the aprismatic layer), dentin or cementum. Regarding the marginal adaptation of the resin-based materials to dentin, it may be compromised by hydrolytic degradation of the hybrid layer [19]. According to other previous studies, when the cervical margins placed in enamel were compared to the ones placed in dentin or cementum, significant differences were recorded, thus in the present study, we chose to assess the microleakage and marginal adaptation at the cervical margin placed in dentin [14,16].

The composition, volume, and weight of the fillers as well as the viscosity and the elasticity modulus of resin-based materials directly influence the shrinkage tension produced during the polymerization process [16,20]. The relation between the shrinkage tension and the adaptation of the materials to cavity walls can be explained by the increased polymerization speed of the outer layers that are closer to the light source, while the internal layers do not benefit from the same polymerization degree [21]. The polymerization reaction can compromise the marginal adaptation of composite resin restorations since only a percentage of 25–50% of the monomer double bonds chemically react, and in the case of an increased C factor (configuration factor-ratio between the bonded and unbonded surfaces) as in the MOD cavities, the unreacted monomers do not have the ability to compensate the polymerization stress, leading to the appearance of marginal microleakage [22].

Regarding the inlay technique, its disadvantages are represented by the additional time required for application, especially in cases when direct restorations can be made and by the difficulty to remove the excess of cementing material from the cervical margins, which could lead to retentive areas formation [23]. Inlays are recommended due to their improved physical and mechanical properties compared to direct restorations [10].

In the present study, we evaluated the marginal sealing of a nanohybrid composite resin applied in mesio-occluso-distal cavities through the direct technique using two different adhesive strategies (etch-and-rinse, respectively, self-etch) and through indirect technique (inlay). The assessment of the marginal adaptation was realized by scanning electron microscopy and optical microscopy at the cervical margins. The obtained results showed that at the cervical margins and the margins relocated using flow composite, the highest microleakage mean values were recorded by the samples restored by the direct technique using the self-etch strategy, while the lowest values were recorded by the samples restored using indirect techniques. These results are not in agreement with the conclusions of a study conducted by Pallesen and Qvist [7], in which inlay restorations showed a lower marginal sealing efficiency compared to direct restorations. Rosa Rodolpho et al. [24] demonstrated through a clinical study an increased efficiency of marginal adaptation of both direct restorations and inlays for small cavities, while for compound or complex cavities, the marginal sealing was not satisfactory.

Another clinical study conducted by Mendonça et al. [23] reported that after a 12-month follow-up period, no significant differences in marginal adaptation at the cervical margin between direct and indirect composite resin restorations were recorded. Therefore, no areas of microleakage were observed, except for a slight discoloration of the material with no signs of secondary caries. These results are in agreement with the conclusions of the studies carried out by Speafico et al. [25] and Wassell et al. [26], in which no differences of marginal adaptation were observed between the two techniques. In another study conducted by Barone et al. [27], no signs of material wear or microleakage were observed at the cervical margin of the inlays. A study performed by Senol et al. showed that no significant differences in microleakage and marginal adaptation were found when comparing MOD indirect vs. direct restorations [16].

At the same time, the obtained results in our study showed that for the cavities restored with inlay technique, no significant differences in marginal adaptation were observed between the cervical margin and the cervical margin relocated with flow composite resin, these results being in agreement with the conclusions of some studies [28,29,30] that found no differences between the marginal sealing at the cervical margins. Fronza et al. [31] reported that the use of flow composite resins did not prevent the appearance of marginal microleakage gaps at the cervical margin. However, several studies have shown that the most effective method of relocating the cervical margin is performed by using flowable resin [32]. Regarding the scanning technique, Cicciu et al. concluded through a systematic review that both digital and analog methods are efficient, thus describing the digital technique as being a more accurate and time saving approach [33].

Regarding the adhesive strategy, in our study, significant differences were observed between etch-and-rinse and self-etch techniques at the margins relocated with flow resin, while at the cervical margins, no differences were found. The obtained results are supported by previous studies [16,28,34,35], in which similar microleakage values were recorded at the cervical margin. Manchorova-Veleva et al. [36] reported that the marginal sealing of composite resins to enamel is more efficient in restorations applied by the etch-and-rinse strategy compared to self-etch. Based on this consideration, we chose in this study to evaluate the dentinal marginal sealing given the reduced sealing quality compared to that of enamel. However, Bhatti et al. [37,38] concluded that none of the adhesive strategies are able to eliminate microleakage gaps at the cervical margins. Tjaderhane et al. [19] reported that regardless of the used adhesive strategy, the portions of collagen that do not bond to the resin become prone to degradation and the quality of the adhesive bond depends on the available substrate. The existence of a damaged dentin-adhesive resin interface may constitute a risk factor for material bonding [19,24].

The obtained results reject the null hypothesis. However, future in vivo and in vitro studies using other evaluation methods such as micro-CT are needed to validate the obtained results. Micro-CT evaluation is a non-destructive method useful in marginal gap measurement [39]. For more clinical relevance, future in vitro studies should be able to reproduce the conditions of the oral environment such as temperature and pH variations, enzymatic and microbial activity, and the mechanics of masticatory movements.

## 5. Conclusions

- Composite resin inlay mesio-occluso-distal restorations present a more effective marginal sealing compared to composite resin applied through the direct technique.
- The adaptation of the materials to cervical margins relocated using flow composite resin is less effective compared to the adaptation at the cervical margins without relocation in mesio-occluso-distal cavities restored by the direct technique, using the etch-and-rinse strategy.
- Relocation of the cervical margins using flow composite resin does not improve marginal adaptation.
- The adhesive strategy in direct restoration technique is not relevant for marginal sealing.

## Figures and Tables

**Table 1:** Application protocol for etchant and adhesive.

Etching Technique	Phosphoric Acid Application	Adhesive Application
Etch-and-rinse	The acid was applied on the exposed dentin for 15 s, then was rinsed for 15 s with water and air-dried for 5 s using the air-spray from the dental unit.	The adhesive was applied on the air-dried surface for 20 s using an adhesive tip applicator with a rubbing action. Then, a slight air pressure was applied for 5 s and the adhesive was light cured for 20 s.
Self-etch	Phosphoric acid was not applied.	

**Table 2:** Name, type, manufacturer, and composition of the tested materials.

Name	Type	Manufacturer	Composition
Miris 2	Nanohybrid composite resin	Coltène Whaledent, Altstaetten, Switzerland	Barium alumino fluoride glass, BisGMA, TEGDMA, UDMA
ScotchBond Universal	Adhesive resin	3M ESPE, St. Paul, MN, USA	Methacryloyloxydecyl, dihydrogen phosphate, phosphate monomer, dimethacrylate resin, hydroxyethyl methacrylate, methacrylate-modified alkenoic acid copolymer, filler, ethanol, water, initiators, silane
Enamel Plus HRi Flow	Flow composite resin	Micerium, Avegno, GE, Italy	BisGMA, UDMA, 1,4-butandiol-dimethacrylate, highly dispersed silicone dioxide 53%vol
Rely X Unicem	Self-adhesive resin cement	3M ESPE, St. Paul, MN, USA	Methacrylated phosphoric acid esters, TEGDMA, substituted dimethacrylate, silanized glass powder, silane treated silica, sodium persulfate, substituted pyrimidine, calcium hydroxide, 72%wt

BisGMA-bisphenol A-glycidyl methacrylate; TEGDMA-Triethylene glycol dimethacrylate; UDMA-Urethane-dimethacrylate.

## Data Availability

All the data presented in this study are available within the article.
